# The prevalence of lower urinary tract symptoms based on individual and clinical parameters in patients with multiple sclerosis

**DOI:** 10.1186/s12883-019-1582-1

**Published:** 2020-01-17

**Authors:** Fatemeh Nazari, Vahid Shaygannejad, Mehrdad Mohammadi Sichani, Marjan Mansourian, Valiollah Hajhashemi

**Affiliations:** 1 Isfahan Neurosciences Research Center, Isfahan University of Medical Sciences, Isfahan, Iran; 2 Department of Adult Health Nursing, Faculty of Nursing and Midwifery, Isfahan University of Medical Sciences, Isfahan, Iran; 3 Department of Neurology, School of Medicine, Isfahan University of Medical Sciences, Isfahan, Iran; 4 Isfahan Kidney Transplantation Research Center, Isfahan University of Medical Sciences, Isfahan, Iran; 5 Department of Urology, School of Medicine, Isfahan University of Medical Sciences, Isfahan, Iran; 6 Department of Epidemiology & Biostatistics, School of Health, Isfahan University of Medical Sciences, Isfahan, Iran; 7 Isfahan Pharmaceutical Sciences Research Center, Isfahan University of Medical Sciences, Isfahan, Iran; 8 School of Pharmacy and Pharmaceutical Sciences, Isfahan University of Medical Sciences, Isfahan, Iran

**Keywords:** Multiple sclerosis, Prevalence, Urinary symptoms

## Abstract

**Background:**

Most patients with multiple sclerosis (MS) suffer from bladder dysfunction during the course of the disease. This study was conducted to examine the prevalence of these complications among patients with MS.

**Methods:**

This cross-sectional study was performed on 602 patients with MS who referred to the neurology clinics of Kashani and Alzahra Hospitals affiliated to Isfahan University of Medical Sciences, Isfahan, Iran. Multistage random cluster sampling was performed and the informed consent form was signed by the subjects. Then, all the data were collected through interviews using the Lower Urinary Tract Symptom Score (LUTSS) developed in accordance with the definitions presented by the International Continence Society (ICS) and the International Prostate Symptom Score (I-PSS) and DASS-21 questionnaire. The data were analyzed using descriptive and inferential statistical tests in SPSS.

**Results:**

The prevalence rate of lower urinary tract symptoms (LUTS) was 87.6% among all the subjects, with a similar rate among women (88.0%) and men (86.0%). There was a significant difference between men and women in terms of the prevalence of stress urinary incontinence (SUI), intermittent urine flow, hesitancy, straining, and dribbling (*P* <  0.050). There was no significant difference between women and men in terms of the prevalence of other symptoms (*P* > 0.050). A significant difference was observed in the degree of LUTS with age, marital status, marriage duration, education, illness duration, clinical course, disability, anxiety, depression, and stress (*P*<  0.05). Moreover, logistic regression analysis revealed that there was a higher probability of a urinary problems among patients with MS and a high EDSS score [0.67 (0.507–0.903), *P* = 0.008].

**Conclusions:**

A high prevalence of LUTS was found among patients with MS. There was a higher probability of a urinary tract problem among patients with MS and a high EDSS score. Therefore, it is recommended that the health system take the necessary measures regarding timely detection and treatment of LUTS among these patients in order to prevent secondary outcomes and improve the quality of life (QOL) of patients with MS.

## Background

In developed countries, multiple sclerosis (MS) is the most prevalent chronic neurological disorder among young individuals, with an incidence rate of more than 400 thousand people in the United States [1] and 500–700 thousand people in Europe [2]. Based on a previous study, the prevalence rate of this disease in Iran is estimated to be between 5.3 and 74.28 per 100 thousand people [3]. Several studies have reported that the majority of patients with MS suffer from lower urinary tract symptoms (LUTS), which are among the most common debilitating social manifestations of the disorder, are often ignored [4], and are a major source of morbidity and financial burden for these patients [5]. Urinary symptoms have been shown to have a detrimental effect on health-related quality of life (HRQOL) in patients with MS. [6]

The rate of urinary complications in patients with MS is significantly higher in comparison to that in the general public [5]. LUTS is a spectrum of symptoms that are subjectively perceived very differently [7]. The European Association of Urology (EAU) and American Urological Association (AUA) guidelines define LUTS as storage (irritative) symptoms (daytime urinary frequency, urgency, and nocturia, urgency incontinence, stress urinary incontinence (SUI), urinary leakage without cause, nocturnal enuresis, and urinary leakage during sexual activity), voiding (obstructive) symptoms (slow stream, intermittency, hesitancy, splitting of stream, straining to void, and terminal dribble), or post micturition symptoms (sensation of incomplete bladder emptying and/or a post micturition dribble) that affect the lower urinary tract (LUT) [8–10]. An overview of the Multiple Sclerosis Association of America (MSAA) showed that 50–65% of patients with MS had a mild disability and complained of at least one moderate to severe LUTS, and LUTS is the initial symptom of the disease in 2 to 14% of patients with MS. [11] The nature of the emptying complaint and LUTS varies between the two sexes in patients with MS. [12] However, there is little information regarding the differences in urinary findings among men and women or subtypes of MS. [13] The results of a study revealed that urinary and urodynamic findings of the two sexes are similar, and despite the similarity in their urodynamic findings, patients with relapsing-remitting MS (RRMS) reported greater LUTS severity compared to patients with secondary progressive multiple sclerosis (SPMS) [14]. Kalsi and Fowler found that obstructive complaints such as urinary hesitancy, discontinuation or weak stream of urine, and incomplete urination have a higher incidence among men. However, the urinary incontinence complaint is more prevalent among women and the prevalence of stimulatory complaints such as urgency, diurnal and nocturnal polyuria, and pain is identical between the sexes [12].

Several studies have shown that some patients consider these symptoms as a manifestation of the disease. Nevertheless, the severity of these symptoms and their impact on the early stage of the disease are not clear [4]. Understanding the characteristics of LUTS may be helpful in the differential diagnosis of various clinical types of MS and in the management of patients with other medical conditions such as stroke, diabetes mellitus (DM), and prostate hypertrophy the effect of which on emptying and sexual function has been recognized. Moreover, such an understanding provides a significant insight into the complicated MS pathophysiology and the mechanism of LUTS in the emergence of MS. These determinants contribute to the management of LUTS and improvement of quality of life (QOL) [15]. Performing epidemiological studies to evaluate the rate of specific symptoms among individuals with MS helps to describe the economic and social significance of the disease and assists in the identification, early diagnosis, and timely control of the complications of the disease [16], and identification of possible causes of the disorder, in addition to providing the foundation for higher level studies [17, 18]. Few studies have examined bladder dysfunction despite its significant societal effects on individuals [19]. Therefore, due to the difference in prevalence of bladder dysfunction in different societies, and the difference observed in the clinical symptoms, emptying dysfunction, and demographic characteristics of patients with MS, and the serious impact of these disorders on QOL, this study was conducted with the aim to determine the prevalence of LUTS based on individual and clinical parameters in patients with MS.

## Methods

This cross-sectional study was conducted on 602 patients with MS from 23rd of August 2017 to 19th of April 2018. The study participants consisted of individuals who referred to the neurology clinic of Kashani and Alzahra Hospitals affiliated to Isfahan University of Medical Sciences, Isfahan, Iran. In this study, the subjects were selected based on multistage random cluster sampling method and the cluster volume was chosen proportional to their population size. The duration of the disease, sex, and the separated number of men and women were considered as the first class, the second class, and clusters, respectively. Using a random number table, the sample size was determined proportional to the size of the men and women clusters. The number of subjects, based on the results of the study of Sand and Sand [20], the prevalence of LUTS (*P* = 0.500), accuracy of 5%, confidence interval (CI) of 95%, d of 0.04, and a dropout rate of 10%, was estimated to be 660. From among the selected participants, 38 and 4 patients were excluded from the study due to their unwillingness to continue cooperation and complete the questionnaire, and due to recurrence of MS and corticosteroid therapy, respectively.

The study inclusion criteria included the definitive diagnosis of MS by a neurologist based on the 2010 McDonald criteria [21], age of above 18 years, having a medical record at a referral clinic, physical and mental ability to answer questions (lack of physical or cognitive defects causing inability to answer the questions correctly), resident of Isfahan City, willingness to participate in the study, stable clinical status [lack of progressive disability or recurrence in the past 3 months based on an increase of at least 1 point in the Expanded Disability Status Scale (EDSS) score], lack of LUTS prior to the onset of MS, lack of history of pelvic surgery/radiotherapy or bladder stones and lack of history of benign prostatic hyperplasia (BPH) or prostate surgery (through patient self-report). In addition, the exclusion criteria included unwillingness to continue cooperation and complete the questionnaire, recurrence of MS in the previous month, and treatment with corticosteroids. After sampling using multistage random cluster sampling method and completion of the informed consent form by the subjects, all the data in this study were obtained through interviews using a three-part questionnaire in a bright, silent room with suitable temperature and ventilation at the MS Clinics of Kashani and Alzahra Hospitals and patients were given rest while filling out the questionnaire. The questionnaire used in this study consists of 4 parts. The first part of the questionnaire contains 6 questions on demographic data including age, sex, marital status, educational status, occupation, and economic status. The second part includes 6 questions on clinical information regarding the duration of the disease, age at the time of diagnosis, EDSS score, type of clinical presentation of MS, current comorbid condition, and type and duration of use of drugs. The third part is the Depression, Anxiety, and Stress Scale (DASS-21) in which each cluster of 7 items measures one factor or emotional state in order to assess depression, anxiety, and stress. Each question is scored on a Likert scale ranging from 0 to 3. Ghafari et al. calculated the internal consistency of the DASS-21 using Cronbach’s alpha and the Cronbach’s alpha of the Subscales of depression, anxiety, and stress was 0.97, 0.71, and 0.74, respectively [22]. The fourth part includes the validated Persian version of the Lower Urinary Tract Symptom Score (LUTSS) [9] and International Prostate Symptom Score (I-PSS) [23]. The LUTSS was not previously used in an Iranian population and was validated through the standard process for the purpose of the present study. The English version of the LUTSS was translated into Persian using forward and backward translation, expert opinions were obtained, and its validity and reliability were assessed in a separate sample through a pilot study. Finally, the reliability and validity of the translated version were confirmed with a Cronbach’s alpha coefficient of 0.789. Khalaf et al. have described the scoring method of the LUTSS [6]. LUTSS contains 18 items that assess the presence of all LUTS, and can be utilized for the comprehensive assessment of LUTS experienced by male and female patients with MS. This questionnaire assesses storage symptoms (8 items: daytime urinary frequency, nocturia, urgency, urgency incontinence, SUI, urinary leakage without cause, nocturnal enuresis, and urinary leakage during sexual activity), voiding problems (6 items: weak stream, intermittency, hesitancy, splitting of stream, straining to void, and terminal dribble), post-micturition (2 items: sensation of incomplete bladder emptying or a post micturition dribble), and other symptoms (2 items: bladder pain, and dysuria). The items are scored based on a 5-point Likert scale [6, 24].

The I-PSS scale includes 7 specific questions associated with urinary and obstructive urinary symptoms and an additional question on general satisfaction with urinary situation that was not used in the scoring of the scale. Polyuria, urgency, and nocturnal polyuria reflected the stimulatory symptoms (maximum score of 15), and incomplete emptying of the bladder, intermittent urine flow, poor urine flow, and straining at the onset of urination were indicative of obstructive symptoms (maximum score of 20). The items were scored based on a 6-point Likert scale ranging from 0 (no problem) to 5 (always), and the symptom scoring was based on the frequency scale ranging from 0 to 5 with a total score range of 0–35 (asymptomatic to very symptomatic). The total score was calculated through adding up the scores of the 7 urinary questions, with scores of 0–7, 8–19, and 20–35 representing a lack of symptoms or presence of mild symptoms, moderate symptoms, and severe symptoms, respectively. In the current study, the Persian version of the I-PSS questionnaire by Panahi et al. was exploited. They showed that with a Cronbach’s alpha value of 0.7, an intra-class correlation coefficient (ICC) of 0.87, and Pearson product-moment correlation coefficient of 0.92, this questionnaire was a valid and reliable scale and could be used as a symptom-based questionnaire in the Iranian population [23]. Moreover, physical disability was rated using the Expanded Disability Status Scale (EDSS) that assesses pyramidal, cerebellar, brainstem, sensory, bowel and bladder, visual, and mental functions. The EDSS was completed (total score: 0–10) for the patients by a neurologist [25]. According to Jones’ criteria, EDSS score of 0–3, 3.5–5.5, and higher than 7 represents mild, moderate, and severe physical disability [26].

The collected data were analyzed using descriptive statistics [mean and standard deviation (SD), percentage, and frequency of variables], and inferential statistics including chi-square test, independent t-test, analysis of variance (ANOVA), and logistic regression analysis test in the SPSS software (version 18, SPSS Inc., Chicago, IL, USA). Moreover, the significance level was considered to be less than 0.05.

## Results

This study included 602 patients with MS. The mean and SD of the age of the participants, duration of MS, age at onset of MS, and disability score were 35.45 ± 9.08 years, 7.63 ± 5.56 years, 26.85 ± 7.81 years, and 2.45 ± 2.11, respectively. According to the EDSS, 432 (71.6%), 151(25.0%), and 20 (3.4%) patients had mild, moderate, and severe disability. In addition, 76.1, 33.7, and 37.8% of the patients had a clinical relapsing-remitting period, treatment with beta interferon’s, and comorbid illnesses, respectively. Moreover, 78.6, 68.8, 40.5, and 21.2% of the subjects were women, were married, had university degrees, and were employed, respectively (Table 1).

**Table 1:** Demographic and Clinical Characteristics of subjects

Parameter	
Age (year) [Mean (SD)]	35.45 (8.08)
Disease Duration [Mean (SD)]	7.63 (5.56)
Age of onset of disease [Mean (SD)]	26.85(7.81)
Sex [n (%)]	
Female	474 (78.7)
Male	128 (21.3)
Marital status [n (%)]	
Single	149 (24.8)
Married	415 (68.9)
Divorced and widowed	38 (6.3)
Education level [n (%)]	
Secondary school or below	145 (24)
high school	214 (35.5)
University	244 (40.5)
Job status [n (%)]	
Employed	128 (21.2)
Unemployed	52 (8.6)
housewife	378 (62.7)
Retired and disabled	45 (7.5)
Current comorbid conditions [n (%)]	
Diabetes	7 (1.2)
Arthritis	8 (1.3)
Hypertension	17 (2.8)
Heart disease	5 (0.8)
Thyroid disorders	40 (6.6)
Migraine	37 (6.1)
Asthma	8 (1.3)
Inflammatory bowel disease	9 (1.5)
kidney disease	14 (2.3)
Liver disease	4 (0.7)
infectious disorders	2 (0.3)
Skin disorders	2 (0.3)
Epilepsy	11 (1.8)
Other disorders	64 (10.6)
None	375 (62.1)
EDSS Score [Mean (SD)]	2.45 (2.11)
EDSS Class [n (%)]	
Mild (1–3)	432 (71.6)
Moderate (3.5–6.5)	151 (25)
Severe (≥ 7)	20 (3.4)
Course disease [n (%)]	
Relapsing remitting	459 (76.1)
Primary progressive	19 (3.2)
Secondary progressive	97 (16.1)
CIS	28 (4.6)
MS medication [n (%)]	
Interferon beta	203 (33.7)
Glatiramer acetate	47 (7.8)
Fingolimod	114 (18.9)
Natalizumab injection	41 (6.8)
Azathioprine	20 (3.3)
Ritoximab injection	97 (16.1)
Mitoxantrone	1 (0.2)
Dimethilfumarat	13 (2.2)
Trifulonumaide	4 (0.7)
Mix (Azathioprine&Interferonbeta)	24 (4)
Cyclophosmaide	8 (1.3)
Metotruxat	3 (0.5)
None	28 (4.6)
Symptomatic treatment [n (%)]	
Anti-depressant	119(19.8)
Anticonvulsants	111(18.4)
Anti-anxiety	40(6.6)
Antipsychotic	17(2.8)
Anticholinergic	103(17.1)
anti-spasticity	86(14.3)
Anti-fatigue	56(9.3)
Beta blocker	37(6.1)
Levothyroxine	26(4.3)
anti-Anemia	35(5.8)
Amantadine	15(2.5)
supplements	377(62.6)
Anti-lipid	8(1.3)
NSAID	10(1.7)
Alpha Adrenergic Inhibitor	16(2.7)
Anti-acid	39(6.5)
Modafinil	8(1.3)

SD: Standard deviation; EDSS: Expanded Disability Status Scale

The prevalence of LUTS was 87.7%, with a similar rate among women (88.2%) and men (85.9%). Furthermore, 18.7% of patients reported experiencing LUTS only in the acute phase and recurrence of the disease. The most commonly reported symptoms among all subjects based on the definitions provided by the International Continence Society (ICS) were nocturnal polyuria (59.3%) and urgency (57.1%) in the storage phase, urinary hesitancy (39.7%) and intermittent urine flow (36.0%) in the emptying phase, and feeling of incomplete emptying in the post-emptying phase (43.2%) (Table 2).

**Table 2:** Prevalence of lower urinary tract symptoms among all the subjects and women and men based on the LUTS Score

LUTS [n (%)]	Total	Women	Men	*P*
	528 (87.7)^a^	418 (88.2)	110(85.9)	0.492**
Storage symptoms				
daytime frequency (Patient who considers that he/she urinates too often by day)	249 (41.4)	203 (42.8)	46 (35.9)	0.160**
Nocturia (Waking up at night one or more times to urinate)	357 (59.3)	289 (61.0)	68 (53.1)	0.109**
Urgency (A sudden compelling desire to urinate)	344 (57.1)	275 (58.0)	69 (53.9)	0.404**
Urgency urinary incontinence (Involuntary leakage of urine accompanied by or immediately preceded by sudden need to rush to urinate)	217 (36.0)	174 (36.7)	43 (33.6)	0.515**
Enuresis (involuntary urination)	19 (3.2)	16 (3.4)	3 (2.3)	0.777***
Stress urinary incontinence (Involuntary leakage of urine on effort or exertion, or on sneezing or coughing)	182 (30.2)	160 (33.8)	22 (17.2)	< 0.001**
Leakage of urine during sexual activity	17 (2.8)	15 (3.2)	2 (1.6)	0.547***
Nocturnal enuresis (Leakage of urine occurring during sleep)	33 (5.5)	24 (5.1)	9 (7.0)	0.385**
Voiding symptoms				
Weak/slow stream (Perception of weak/reduced urine flow, usually compared with previous performance)	171 (28.4)	131 (27.6)	40 (31.3)	0.421**
Split stream (Splitting or spraying of the urine stream may be reported)	159 (26.4)	129 (27.2)	30 (23.4)	0.390**
Intermittent urine stream (Urine flow stops and starts on one or more occasions during micturition)	217 (36.0)	160 (33.8)	57 (44.5)	0.024**
Hesitancy (Difficulty in initiating urination resulting in a delay in the onset of urination after the individual is ready to pass urine)	239 (39.7)	169 (35.7)	70 (54.7)	< 0.001**
Straining (Muscular effort used to either initiate, maintain, or improve the urinary stream)	153 (25.4)	106 (22.4)	47 (36.7)	0.001**
Terminal dribble (A trickle or dribble at the end of the urine flow)	64(10.6)	44(9.3)	20(15.6)	0.39**
Post-micturition symptoms				
Feeling of incomplete emptying (Feeling that the bladder is not empty after urination)	260 (43.2)	202 (42.6)	58 (45.3)	0.585**
Post-micturition dribble (Involuntary loss of urine immediately after an individual has finished passing urine	171 (28.4)	124 (26.2)	47 (36.7)	0.019**
Other genitourinary symptoms				
Bladder pain (Pain or discomfort in the bladder area)	106 (17.6)	84 (17.7)	22 (17.2)	0.880**
Dysuria (Burning sensation during urination)	88 (14.6)	64 (13.5)	24 (18.8)	0.136**

*LUTS* Lower urinary tract symptoms; ^a^ Of the 602 participants, 528 (87.7%) reported some type of LUTS; Data was presented as frequency (percentage), ** *P*-values were derived from chi-square test.). *** *P*-values were derived from Fisher’s exact test

Symptoms with the highest prevalence among women were, respectively, nocturia (61.0%) and urgency (58.0%) in the storage phase, hesitancy (35.7%) and intermittent urine flow (33.8%) in the emptying phase, and feeling of incomplete emptying in the post-emptying phase (42.6%). Among men, symptoms with the highest prevalence included urgency (53.9%) and nocturia (53.1%) in the storage phase, hesitancy (54.7%) and intermittent urine flow (44.5%) in the emptying phase, and feeling of incomplete urine emptying in the post-emptying phase (45.3%). Symptoms of bladder pain and burning sensation while urinating were common among 17.7 and 18.8% of women and men, respectively. The prevalence of SUI symptoms was reported as 33.8 and 17.2% among women and men, respectively, and the chi-square test showed a significant difference between the two groups (*P* <  0.001). Moreover, this test showed a significant difference between the two groups of men and women in terms of the prevalence of intermittent urine flow, urinary hesitancy, straining, and dribbling (*P* <  0.050). There was no significant difference between the two groups of men and women in terms of the prevalence of other symptoms (*P* > 0.050) (Table 2).

The prevalence of the times Intermittent urine stream, frequency, and straining at the onset of urination symptoms was not similar between men and women, and the chi-square test indicated significant differences between the two groups (*P*<  0.050). However, there was no significant difference between the two groups of men and women in terms of the prevalence of other symptoms (*P*> 0.050) (Table 3).

**Table 3:** Comparison of prevalence of times lower urinary tract symptoms among women and men with multiple sclerosis based on the International Prostate Symptom Score (I-PSS)

I-PSS	Sex	Not at all [n (%)]	Less than half the time [n (%)]	More than half the time [n (%)]	*P*
Urgency	Women	200 (42.2)	146 (30.8)	128 (27.0)	0.416
	Men	59 (45.7)	32 (24.8)	38 (29.5)	
Frequency	Women	272 (57.4)	52 (11.0)	150 (31.6)	0.048
	Men	82 (63.6)	5 (3.6)	42 (32.6)	
Nocturia	Women	185 (39.0)	247 (52.1)	42 (8.9)	0.230
	Men	61 (47.3)	59 (45.7)	9 (7.0)	
Feeling of incomplete emptying	Women	272 (57.4)	122 (25.7)	80 (16.9)	0.687
	Men	71 (55.0)	32 (24.8)	26 (20.2)	
Intermittent urine stream	Women	314 (66.2)	74 (15.6)	86 (18.1)	0.011
	Men	72 (55.8)	18 (14.0)	39 (30.2)	
Straining	Women	368 (77.6)	49 (10.3)	57 (12.0)	0.001
	Men	82 (63.6)	16 (12.4)	31 (24.0)	
Weak/slow stream	Women	343 (72.4)	63 (13.3)	68 (14.3)	0.360
	Men	89 (69.0)	15 (11.6)	25 (19.4)	

*I-PSS* International Prostate Symptom Score; Data was presented as frequency (percentage). P-values were derived from chi-square test

The prevalence of mild, moderate, and severe LUTS was 46.3, 24.5, and 5.8% in the 18–30 years age group, 44.9, 33.6, and 8.1% in the 30–40 years age group, and 37.1, 40.7, and 12.2% in the 40–50 years age group, respectively. The chi-square test showed a significant difference between the four groups in this regard (*P* <  0.001). Furthermore, the mean age of the patients with mild, moderate, and severe LUTS was 34.50 ± 7.87, 37.51 ± 7.98, and 37.60 ± 8.33 years, respectively, and ANOVA showed a significant difference among LUTS severity groups in terms of age (*P* ≤ 0.001).

The mean body mass index (BMI) of the patients with mild, moderate, and severe LUTS was 24.22 ± 4.25, 24.31 ± 4.51, and 23.93 ± 4.16 Kg/m^2^, respectively, and ANOVA showed no significant difference among LUTS severity groups in terms of BMI (*P* = 0.670). The prevalence of mild, moderate, and severe LUTS was, respectively, 44.3, 33.8, and 6.7% among women and 39.1, 28.9, and 14.8% among men, and the chi-square test indicated a significant difference between the two groups (*P* = 0.024). This rate was 40.9, 29.6, and 8.7% among single individuals, 45.1, 33.0, and 7.4% among married subjects, and 31.6, 42.1, and 18.4% among divorced and dead subjects, respectively, and the chi-square test showed significant differences among the three groups (*P* = 0.051). Furthermore, the mean duration of marriage of the patients with mild, moderate, and severe LUTS was 13.57 ± 8.24, 18.33 ± 8.76, and 15.65 ± 10.01 years, respectively, and ANOVA showed a significant difference among LUTS severity groups in terms of mean duration of marriage (*P* <  0.001). The prevalence of mild, moderate, and severe LUTS was, respectively, 32.4, 48.3, and 11.0% in the group with secondary school degree or lower, 51.5, 28.0, and 6.5% in the group with a high school degree, and 42.2, 27.5, and 8.6% in the group with a university degree, and the chi-square test showed a significant difference among the three groups (*P* <  0.001) (Table 4).

**Table 4:** Comparison of prevalence of lower urinary tract symptoms in terms of individual and clinical characteristics based on the International Prostate Symptom Score (I-PSS)

Age group (year) [n (%)]	No symptoms	Mild (1–7)	Moderate(8–19)	Severe (20–35)	*P*
18–30	44 (23.4)	87 (46.3)	46 (24.5)	11 (5.8)	< 0.001*
30–40	33 (13.4)	111 (44.9)	83 (33.6)	20 (8.1)	
40–50	17 (10.2)	62 (37.1)	68 (40.7)	20 (12.0)	
Age (year) [Mean (SD)]	32.63 (7.48)	34.50 (7.87)	37.51 (7.98)	37.60 (8.33)	< 0.001**
BMI (kg/m^2^)	23.69(3.88)	24.22(4.25)	24.31(4.51)	23.93(4.16)	0.670**
Sex n (%)					
Female	72 (15.2)	210 (44.3)	160 (33.8)	32 (6.7)	0.024*
Male	22 (17.1)	50 (39.2)	37 (28.9)	19 (14.8)	
Marital status [n (%)]					
Single	31 (20.8)	61 (40.9)	44 (29.6)	13 (8.7)	0.051*
Married	60 (14.5)	187 (45.1)	137 (33)	31 (7.4)	
Divorced and widowed	3 (7.9)	12 (31.6)	16 (42.1)	7 (18.4)	
Duration of marriage (year)	12.34(8.09)	13.57(8.24)	18.33(8.76)	15.65(10.01)	< 0.001**
Education level [n (%)]					
Secondary school or below	12 (8.3)	47 (32.4)	70 (48.3)	16 (11)	< 0.001*
High school	29 (13.6)	110 (51.6)	60 (28.2)	14 (6.6)	
University	53 (21.7)	103 (42.2)	67 (27.5)	21 (8.6)	
Disease duration (year) [n (%)]					
< 5	45 (19.5)	115 (49.8)	62 (26.8)	9 (3.9)	< 0.001*
5–10	34 (16.1)	91 (43.2)	71 (33.6)	15 (7.1)	
> 10	15 (9.4)	54 (33.8)	64 (40.0)	27 (16.8)	
EDSS (Expanded Disability Status Scale) [n (%)]					
1–3	85 (19.5)	212 (49.2)	112 (26.0)	23 (5.3)	< 0.001*
3.5–6.5	7 (4.6)	43 (28.5)	76 (50.5)	25 (14.6)	
≤ 7	3 (15)	5 (25)	9 (45)	3 (15)	
Course disease [n (%)]					
Relapsing remitting	79 (17.3)	215 (46.9)	132 (28.8)	32 (7.0)	< 0.001*
Progressive	6 (5.2)	31 (26.7)	60 (51.7)	19 (16.4)	
CIS	9 (32.1)	14 (50.0)	5 (17.9)	0 (0.0)	
Anxiety [Mean (SD)]	10.59(8.36)	13.28(9.07)	15.34(9.24)	15.37(8.10)	< 0.001**
Depression [Mean (SD])	11.00(8.78)	13.70(9.30)	17.25(10.09)	16.35(8.44)	< 0.001**
Stress [Mean (SD)]	17.72(10.43)	19.63(10.25)	21.97(9.78)	20.82(8.04)	0.005**

*EDSS* Expanded Disability Status Scale; CIS: Clinically isolated syndrome; Values are means ± SD; *P-value resulted from Chi-Square and **P-value resulted from ANOVA Tests

The prevalence rate of mild, moderate, and severe LUTS was 49.8, 26.8, and 3.9% in the group with duration of illness of < 5 years, 43.2 33.6, and 7.1% in the group with duration of illness of 5–10 years, and 33.8, 40.0, and 16.8% in the group with duration of illness of > 10 years, respectively. The chi-square test revealed a significant difference among the three groups in this respect (*P* <  0.001). In addition, this rate was 49.2, 26.0, and 5.3% in the group with mild disability, 28.5, 50.5, and 14.6% in the group with moderate disability, and 25.0, 45.0, and 15.0% in the group with severe disability, respectively, and the chi-square test indicated a significant difference among the three groups (*P* <  0.001). Finally, the prevalence of mild, moderate, and severe LUTS was 50.0, 17.9, and 0.0% in the group with clinically isolated syndrome (CIS), 46.9, 28.8, and 7.0% in the group with RRMS, and 26.7, 51.7, and 16.4% in the group with a progressive clinical course, respectively, and the chi-square test showed a significant difference among the three groups (*P* <  0.001).

The mean anxiety score of the patients with mild, moderate, and severe LUTS was 13.70 ± 9.30, 17.25 ± 10.09, and 16.35 ± 8.44, respectively, and ANOVA showed a significant difference among LUTS severity groups in terms of mean anxiety score (*P*<  0.001) The mean depression score of the patients with mild, moderate, and severe LUTS was 13.28 ± 9.07, 15.34 ± 9.24, and 15.37 ± 8.10, respectively, and ANOVA showed a significant difference among LUTS severity groups in terms of mean depression score (*P*<  0.001). The mean stress score of patients with mild, moderate, and severe LUTS was 19.63 ± 10.25, 21.97 ± 9.78, and 20.82 ± 8.04, respectively, and ANOVA showed a significant difference Among LUTS severity groups in terms of mean stress score (*P*<  0.001) (Table 4).

Moreover, variables that showed significant unadjusted associations with urinary problems were age, marital status, marriage duration, educational status, disease duration, EDSS, clinical course of the disease, anxiety, depression, and stress. The final regression model showed that there was a higher probability of a urinary problem among patients with MS and a high EDSS score [0.67(0.507–0.903); *P* = 0.008] (Table 5).

**Table 5:** Multivariable logistic regression analysis of patients with multiple sclerosis and urinary problems

Characteristic	Unadjusted odds ratio (95% CI)	P	Adjusted odds ratio (95% CI)	P*
Age (year)	0.938 (0.907 to 0.969)	< 0.001	0.916 (0.766–1.097)	0.342
Marital status	1.400 (0.845 to 2.320)	0.004	3.049 (0.379–24.545)	0.295
Gender				
Female	0.819 (0.463 to 1.449)	0.492	0.499 (0.198–1.259)	0.141
Male	Ref			
Marriage duration (year)	0.947 (.912 to 0.983)	0.005	1.014 (0.937–1.097)	0.724
Educational status				
Secondary school degree or below	0.338 (0.159 to 0.718)	0.005	0.573 (0.199–1.649)	0.301
High school degree	0.678 (0.396 to 1.161)	0.157	0.748 (0.353–1.586)	0.449
University degree	Ref			
Age at onset of disease (year)	0.979 (0.948 to 1.011)	0.187	1.058 (0.902–1.242)	0.486
Disease duration (year)	0.922 (0.876 to 0.972)	0.002	1.063 (0.894–1.263)	0.489
EDSS	0.647 (0.545 to 0.768)	< 0.001	0.677 (0. 507–0.903)	0.008
Course of the disease				
RRMS	0.496 (0.203 to 1.214)	0.125	0.358 (0.118–1.090)	0.071
Progressive	0.053 (0.010 to 0.271)	< 0.001	0.000 (0.000-)	0.996
CIS	Ref			
Anxiety	0.950 (0.923 to 0.979)	0.001	0.974 (0.913 to 1.039)	0.429
Depression	0.934 (0.906 to 0.962)	< 0.001	0.945 (0.886 to 1.007)	0.080
Stress	0.965 (0.941 to 0.990)	0.006	1.031 (0.972 to 1.093)	0.307

*EDSS* Expanded Disability Status Scale, *RRMS* Relapsing-remitting multiple sclerosis, *CIS* Clinically isolated syndrome

OR adjusted based on individual variables (age, sex, marital status, duration of marriage, educational status, and occupation) and clinical variables (age at onset of disease, disease duration, EDSS, course of the disease, anxiety, depression, and stress)

## Discussion

MS is a demyelination disease of the central nervous system (CNS) that causes impairment of conduction velocity in axonal pathways. This impairment causes several neurological abnormalities including urological dysfunctions. The symptomatology of MS ranges widely according to the location of lesions in the CNS [27]. MS plaque location is a key feature in the pathophysiology of disease-related LUTS [28]. The unstable pattern and location of the demyelination area along with the associated edema are responsible for the alteration in both the neurologic and urologic features of MS. In general, suprasacral plaques will cause varying degrees of detrusor hyperreflexia, and sacral plaques will result in detrusor hypocontractility. The wide range of LUTS in MS patients is related to the disease characteristic. The demyelinating process can be seen in every part of the CNS from lateral corticospinal columns to the lumbosacral cord [29]. The findings of this cross-sectional study were indicative of the high prevalence of LUTS among patients with MS. Overall, 87.6% of patients reported LUTS and no significant difference was seen between men and women. In this regard, the overall prevalence of LUTS in the studies by Khalaf et al. [6], Onal et al. [30], and Nakipoglu et al. [31] was, respectively, 92.0, 93.0, and 80.8%. Khalaf et al. reported a similar prevalence among women (93.0%) and men (91.0%) [6]. Moreover, a review study revealed that LUTS were common among patients with MS and 80–100% of patients suffer from LUTS during the course of MS. [32]

In the present study, the highest rates of LUTS reported among all of the subjects were related to nocturia, urgency, diurnal polyuria, and feeling of incomplete urine emptying, respectively. A study on 9700 patients with MS revealed that nocturia and urgency were the first and second most prevalent LUTS in these patients [28]. In a study, the incidence of polyuria, straining, nocturnal polyuria, urgency, urinary incontinence, urinary discontinuation, and feeling of incomplete urine emptying among patients with MS was reported to be 61.9, 50.0, 47.6, 47.6, 38.0, 30.9, and 28.5%, respectively [33]. In several studies, the incidence of urgency, polyuria, urgent incontinence, and urination hesitance was reported, respectively, to be 24.0–86.0%, 17.0–65.0%, 34.0–72.0%, and 25.0–49.0% among patients with MS and LUTS [34]. The findings of other investigations showed that the most prevalent symptoms were dribbling (64.0%), urgency (62.0%), and feeling of incomplete emptying (61.0%) [10]. However, the rate of dysuria in the current study (14.6%) was similar to that in the study by Nakipoglu et al. (13.0%) [31], but was lower in comparison to the studies by Goldstein et al. (28.0%) [35], and Wheeler et al. (36.0%) [36].

A review study showed that the storage symptoms of urgency and nocturia were the main symptoms of LUTS, with incidence rates of higher than 50.0% among the subjects, and the prevalence of poor urine flow (50.0%), urinary discontinuation (46.6%), and feeling of incomplete urinary emptying (43.3%) was higher [37]. Overall, previous reports in western countries indicate that stimulatory symptoms (storage phase) are significant urethral symptoms, whereas the incidence of obstructive symptoms (emptying phase) is higher than that of stimulatory symptoms in the east [38].

In the present study, the nature of the emptying complaints and LUTS in patients with MS varied between the two sexes, so that the most prevalent symptoms reported among women were nocturnal polyuria, followed by urgency, and incomplete urine emptying, and SUI with a rate of 33.5% was more prevalent among women compared to men. There was a significant statistical difference between the two groups. Massot et al. reported that SUI was the most prevalent type of incontinence among women, with a prevalence of 31.4% [39] and 16.0% in Dillon et al. study [40].

In the present study, the most prevalent symptoms reported in men included urgency, urinary hesitancy, nocturia, and feeling of incomplete urinary emptying, respectively. Moreover, the incidence of symptoms of intermittent urine flow, urinary hesitancy, straining, and dribbling among men was higher in comparison to women and there was a significant difference between the two groups. In this regard, Gündoğdu et al. found that obstructive complaints such as urinary hesitancy, discontinuity or poor flow of urine, and incomplete urinary emptying were observed most often among men, whereas SUI complaints were more common among women [33]. They found stimulatory complaints including urgency, diurnal and nocturnal polyuria, and pain in both sexes [33]. A study in Japan reported that polyuria was the most prevalent symptom among men (94.8%), followed by a poor flow (92.0%), diurnal polyuria (88.2%), and urgency (70.0%) [41].

In the current study, the frequency of symptoms of intermittent urinary flow and straining at the onset of urination were more prevalent among men compared to women more than half the time, whereas the frequency of diurnal polyuria symptom was higher in women than men less than half the time and the difference was significant. The study by Dillon et al. indicated that urinary symptoms in the emptying phase were more prevalent in patients with MS and LUTS [40].

The results of the current study showed that the mean age of the subjects with severe symptoms was higher in comparison to those with mild and moderate symptoms. In addition, the prevalence of mild urinary symptoms was higher in the age group of below 40 years, whereas the prevalence of moderate and severe LUTS among patients with MS was higher in the age group of above 40 years. In general, with increasing age, the incidence of moderate and severe LUTS increased in patients with MS. Coyne et al. found that the incidence of LUTS increases with age among men and women [42]. The incidence of the urgency and nocturnal polyuria symptoms was also found to increase with increasing age [41].

In this study, the prevalence of severe LUTS among men was higher compared to women. In this regard, in a study, 79.0% of men and 49.0% of women were reported to have moderate to severe symptoms based on the I-PSS scale [4]. Moreover, in the current study, the prevalence of moderate and severe LUTS among individuals with a secondary school degree or below was higher than those with high school and university degrees, and there was a significant difference between the individual variables of age, sex, marital status, and severity of urinary symptoms in this regard.

Furthermore, in this study, the estimated prevalence of severity of LUTS varied depending on the duration of the disease, severity of disability, and clinical course of the disease. With increased duration of the disease for more than 10 years, the prevalence of moderate and severe LUTS increased in patients with MS. Moreover, the prevalence of severe LUTS among patients with MS and severe disability was higher than those with mild to moderate disability. In this regard, the results of a study showed that the prevalence of LUTS increased with increase in the duration of the disease, as an average of 35.0–39.0% of the patients reported the onset of the disease after 5–6 years; in contrast, 64% of the patients with a 17.1-year history of the disease reported LUTS [1, 11]. However, the study by Khalaf et al. indicated that despite the increasing incidence of LUTS with the progress of the disease, these symptoms were not basically related to the duration of the disease or degree of disability [10]. In addition, in the study by Nakipoglu et al., there was no correlation between LUTS and characteristics of the disease [31]. It was found in another study that disability and LUTS were correlated (r = 0.24, *P* = 0.090) [4].

Furthermore, in the current study, the prevalence of moderate and severe LUTS among patients with SPMS was higher than those with CIS and RRMS. In the same vein, in a study by UKKONEN, it was found that patients with progressive MS had a higher prevalence of urinary disorders in comparison to other clinical patterns [35]. In addition, the study by Cox et al. showed that, despite the similarity of urodynamic findings, patients with RRMS reported more severe urinary symptoms in comparison to those with SPMS [14]. The results of the current study showed that the mean anxiety score of the subjects with severe symptoms was higher in comparison to those with mild and moderate symptoms. The mean depression score of the subjects with moderate symptoms was higher in comparison to those with mild and severe symptoms. In addition, the mean stress score of the subjects with moderate symptoms was higher in comparison to those with mild and severe symptoms. In this regard, Coyne et al. showed that chronic anxiety and depression had a significant association with urinary symptoms [42]. Moreover, logistic regression analysis revealed that there was a higher probability of a urinary problem among patients with MS and a high EDSS score [0.67 (0.507–0.903), *P* = 0.008].

The strengths of the present study included the appropriate sample size and observance of the women to men population ratio based on the ratio of MS prevalence within the society. The major limitation of the present study is that as our main results were based on patients’ memories regarding LUTS during the course of their disease; thus, there could be some recall bias in the reported symptoms. Another possible limitation of the present study is the patients’ subjective perception of the investigated LUTS and the lack of objective urodynamic assessments and not necessarily at the time of study participation, and thus the results’ lack of representation of the investigated population. In addition, MRI evaluation could not be applied to patients. Our observation suggests that further studies need to be performed with more advanced techniques.

## Conclusion

A high prevalence of LUTS was found among patients with MS, with similar prevalence rates among men and women. However, the nature of emptying complaints and LUTS differed among women and men with MS, so that the prevalence of intermittent urine flow, urinary hesitancy, straining, and dribbling was higher in men in comparison to women. In addition, the prevalence of severe LUTS in men was higher than women; however, SUI was more prevalent among women than men. There was a correlation between the individual variables (age, sex, marital status, and occupation) and the severity of LUTS. Furthermore, the estimated prevalence of the severity of LUTS varied depending on the duration of the disease, severity of disability, and the clinical course of the disease. Thus, a higher prevalence of moderate and severe LUTS was observed among patients with SPMS in comparison to those with other clinical patterns; in addition, with increasing duration of the disease and severity of disability, the prevalence of moderate and severe LUTS increased in patients with MS. In addition, patients with MS and a high EDSS score were at greater risk of a urinary tract disorder. Therefore, given the high prevalence of LUTS in patients with MS, it is recommended that the health system take the necessary measures regarding the timely detection and treatment of LUTS among these patients. The identification and timely diagnosis of these cases can greatly contribute to the prevention of secondary outcomes and improvement of the QOL of patients with MS.

## Data Availability

The datasets generated and/or analyzed during the current study are not publicly available due but are available from the corresponding author on reasonable request.

## Abbreviations

EDSS: Expanded Disability Status Scale
IPSS: International Prostate Symptom Score
LUTS: Lower urinary tract symptoms
MRI: Magnetic resonance imaging
MS: Multiple sclerosis
MSAA: Multiple Sclerosis Association of America
QOL: Quality of life
QOL: Quality of life
SPMS: Secondary progressive MS
SUI: Stress urinary incontinence

## References

[1] Pintér A, Cseh D, Sárközi A, Illigens B, Siepmann T (2015). Autonomic dysregulation in multiple sclerosis. Int J Mol Sci.

[2] Bevan S, Steadman K (2015). Multiple Sclerosis & Employment in Europe.

[3] Etemadifar M, Sajjadi S, Nasr Z, Firoozeei TS, Abtahi SH, Akbari M (2013). Epidemiology of multiple sclerosis in Iran: a systematic review. Eur Neurol.

[4] Nortvedt M, Riise T, Frugaård J, Mohn J, Bakke A, Skår A (2007). Prevalence of bladder, bowel and sexual problems among multiple sclerosis patients two to five years after diagnosis. Mult Scler J.

[5] Sammarco Anne G, Bogdan O, Mahajan Sangeeta T (2014). The bladder in MS: a review. Neurol Neurophysiol.

[6] Khalaf KM, Coyne KS, Globe DR, Armstrong EP, Malone DC, Burks J (2015). Lower urinary tract symptom prevalence and management among patients with multiple sclerosis. Int J MS care.

[7] Vishwajit S, Andersson KE (2009). Terminology of lower urinary tract symptoms. Helpful or confusing?. Sci World J.

[8] Park HJ, Won JE, Sorsaburu S, Rivera PD, Lee SW (2013). Urinary tract symptoms (LUTS) secondary to benign prostatic hyperplasia (BPH) and LUTS/BPH with erectile dysfunction in Asian men: a systematic review focusing on tadalafil. World J Men's Health.

[9] Abrams P, Cardozo L, Fall M (2002). The standardization of terminology of lower urinary tract function: report from the standardisation sub-committee of the international continence society. NeurourolUrodyn.

[10] Khalaf KM, Coyne KS, Globe DR, Malone DC, Armstrong EP, Patel V (2016). The impact of lower urinary tract symptoms on health-related quality of life among patients with multiple sclerosis. NeurourolUrodyn.

[11] Tracey JM, Stoffel JT (2016). Secondary and tertiary treatments for multiple sclerosis patients with urinary symptoms. Investig Clin Urol.

[12] Kalsi V, Fowler CJ (2005). Therapy insight: bladder dysfunction associated with multiple sclerosis. Nat Clin Pract Urol.

[13] Mahajan ST, Patel PB, Marrie RA (2010). Under treatment of overactive bladder symptoms in patients with multiple sclerosis: an ancillary analysis of the NARCOMS patient registry. J Urol.

[14] Cox L, Cameron AP, Wittman D, Papin JE, Mao-Draayer Y (2015). Analysis of urinary symptoms and urodynamic findings in multiple sclerosis patients by gender and disease subtype. J Neurol Neurobiol.

[15] Wang T, Huang W, Zhang Y (2016). Clinical characteristics and urodynamic analysis of urinary dysfunction in multiple sclerosis. Chin Med J (Engl).

[16] Bahareh S-N, Tannaz R, Arash B, Farid N, Abbas A (2013). Epidemiological characteristics of patients with multiple sclerosis in Kermanshah, Iran in 2012. J Mazand Univ Med Sci.

[17] Ebers GC (2008). Environmental factors and multiple sclerosis. Lancet Neurol.

[18] Hojjati S, Zarghami A, Yousefzad T, Hojjati S, Baes M (2016). Epidemiological features of 263 patients with multiple sclerosis residing in Babol, Iran. J Babol Univ Med Sci.

[19] Hennessey A, Robertson N, Swingler R, Compston D (1999). Urinary, faecal and sexual dysfunction in patients with multiple sclerosis. J Neurol.

[20] Sand PK, Sand RI (2013). The diagnosis and management of lower urinary tract symptoms in multiple sclerosis patients. Disease-a-Month: DM.

[21] Polman CH, Reingold SC, Banwell B, Clanet M, Cohen JA, Filippi M (2011). Diagnostic criteria for multiple sclerosis: 2010 revisions to the McDonald criteria. Ann Neurol.

[22] Ghafari S, Ahmadi F, Nabavi M, Memarian R (2008). The effects of progressive muscle relaxation on depression anxiety and stress in multiple sclerosis patients. J Med.

[23] Ali P, Reza B, Darab M, Omid R (2013). Validity and reliability of Persian version of international prostate symptom score. GMJ.

[24] Blaivas JG, Tsui JF, Mekel G, Benedon MS, Li B, Friedman FM (2015). Validation of the lower urinary tract symptom score. Can J Urol.

[25] Kurtzke JF (1983). Rating neurologic impairment in multiple sclerosis: an expanded disability status scale (EDSS). Neurology.

[26] Collins CD, Ivry B, Bowen JD, Cheng EM, Dobson R, Goodin DS (2016). A comparative analysis of patient-reported expanded disability status scale tools. Mult Scler.

[27] Williams David (2012). Management of bladder dysfunction in patients with multiple sclerosis. Nursing Standard.

[28] Aharony SM, Lam O, Corcos J (2017). Evaluation of lower urinary tract symptoms in multiple sclerosis patients: review of the literature and current guidelines. Can Urol Assoc J.

[29] Akgül M, Yazıcı C, Turgut N, Malak A, Şahin MF (2019). An unexpected lower urinary tract symptom of multiple sclerosis: Valsalva induced urge incontinence. Clin Med Rev Case Rep.

[30] Onal B, Siva A, Buldu I, Demirkesen O, Cetinel B (2009). Voiding dysfunction due to multiple sclerosis: a large scale retrospective analysis. Int Braz J Urol.

[31] Nakipoglu G, Kaya A, Orhan G, Tezen O, Tunc H, Ozgirgin N (2009). Urinary dysfunction in multiple sclerosis. J Clin Neurosci.

[32] Tubaro A, Puccini F, De Nunzio C, Digesu G, Elneil S, Gobbi C (2012). The treatment of lower urinary tract symptoms in patients with multiple sclerosis: a systematic review. Curr Urol Rep.

[33] Gündoğdu AA, Kotan D, Atik YT, Sayan S, Sağlam HS (2016). Lower urinary tract dysfunction in multiple sclerosis patients. Sakarya Med J.

[34] Betts CD, D’Mellow MT, Fowler CJ (1993). Urinary symptoms and the neurological features of bladder dysfunction in multiple sclerosis. J Neurol Neurosurg Psychiatry.

[35] Goldstein I, Siroky MB, Sax DS, Krane RJ (1982). Neurourologic abnormalities in multiple sclerosis. J Urol.

[36] Wheeler JS, Siroky MB, Pavlakis AJ (1983). The changing urologic pattern of multiple sclerosis. J Urol.

[37] Litwiller SE, Frohman EM, Zimmern PE (1999). Multiple sclerosis and the urologist. J Urol.

[38] Araki I, Matsui M, Ozawa K, Nishimura M, Kuno S, Saida T (2002). Relationship between urinary symptoms and disease-related parameters in multiple sclerosis. J Neurol.

[39] Massot C, Khenioui H, Agnani O, Guyot M-A, Hautecoeur P, Donze C (2016). Stress urinary incontinence in women with multiple sclerosis. Int Neurourol J.

[40] Dillon BF, Seideman CA, Lee D, Greenberg B, Frohman EM, Lemack GE (2013). A surprisingly low prevalence of demonstrable stress urinary incontinence and pelvic organ prolapse in women with multiple sclerosis followed at a teriary nerogenic bladder clinic. J Urol.

[41] Yamaguchi O, Aikawa K, Shishido K, Nomiya M (2009). Place of overactive bladder in male lower urinary tract symptoms. World J Urol.

[42] Coyne KS, Kaplan SA, Chapple CR, Sexton CC, Kopp ZS, Bush EN (2009). Risk factors and comorbid conditions associated with lower urinary tract symptoms: EpiLUTS. BJU Int.

